# Full-endoscopic uniportal retropharyngeal odontoidectomy: A preliminary case report

**DOI:** 10.3389/fsurg.2022.973064

**Published:** 2023-01-06

**Authors:** Jichao Ye, Bin Liu, Jinteng Li, Guan Zheng, Kaidi Duan, Liangbin Gao, Chunyan Zhang, Jingwen Huang, Yong Tang

**Affiliations:** ^1^ Department of Orthopedics, Sun Yat-sen Memorial Hospital of Sun Yat-sen University, Guangzhou, China; ^2^ Department of Orthopedics, Lishui People's Hospital, Li Shui, China; ^3^ Department of Orthopedics, The Eighth Affiliated Hospital of Sun Yat-sen University, Shenzhen, China; ^4^ Department of Surgery Center, Sun Yat-sen Memorial Hospital of Sun Yat-sen University, Guangzhou, China; ^5^ Department of Orthopedics, The Second Affiliated Hospital of Guilin Medical University, Guilin, China

**Keywords:** basilar invagination, axis, endoscopy, odontoidectomy, trans-cervical approach

## Abstract

**Summary of background data:**

Odontoidectomy aims to decompress the medulla oblongata and is usually performed through the classical transoral approach, which affects oropharynx and accompanied with high rate of complications comprising swallowing and respiratory tract. We have developed a minimal invasive method *via* a standard cervical anterior approach: full-endoscopic trans-cervical odontoidectomy, which provides an alternative access for the resection of odontoid process and medulla oblongata decompression without traversing potentially contaminated cavities.

**Methods:**

From 2018 to 2020, three patients with either odontoid process lesion or basilar invagination underwent full-endoscopic uniportal trans-cervical odontoidectomy with/without combining the posterior instrumentation. With fluoroscopic guidance, a uniportal endoscope sleeve was placed inside of the odontoid process; then odontoid process was gradually resected from the inside to outside under endoscopic monitoring. Postoperative images and clinical data were collected during post-op follow-up.

**Result:**

Patients were soon extubated after surgery when patients wake up from general anesthesia. There were no severely perioperative complications, especially dysphagia and airway obstruction, and the symptoms and neurological function was improved immediately after surgery. The final pathology of one patient with odontoid osteolytic lesion was confirmed as plasmacytoma. The postoperative CT scans proved that the range of odontoid process resection was consistent with the preoperative expectation.

**Conclusion:**

In summary, our proposed endoscopic trans-cervical odontoidectomy provides a valid choice for non-oral approach, which would reduce postoperative approach related complications and accelerate postoperative recovery.

## Introduction

Odontoidectomy is necessary in cases of irreducible spinal cord compression induced by dislocated odontoid process or odontoid process lesion. Transoral approach odontoidectomy remains to be the “gold standard” in the literature (1, 2). Progress in surgical technique and improvement of understanding anatomic characteristics in this region has deceased the complications and mortality of odontoidectomy. However, there is still a high rate of complications related to throat dysfunction in odontoidectomy via transoral approach due to its damage to mucosal sensory receptors (1, 3, 4). Sensory receptors in the mucosa of pharynx and larynx are essential to the pharyngeal reflex. Attenuation or lack of pharyngeal reflex would increase the opportunity of postoperative aspiration due to post-op bleeding, secretions, and gastric contents. At the same time, severe pharyngeal and laryngeal edema may cause perioperative asphyxia. In addition, bacterial colonization and non-effective preoperative disinfection of the pharyngeal cavity give rise to the incidence of postoperative infection (3, 4).

To avoid the damage to pharynx and larynx mucosa in bypassing these cavities, surgeons had been attempting to apply a trans-cervical approach in odontoidectomy. A trans-cervical retropharyngeal exposure of the odontoid process was reported by Fong and DuPlessis (5). The approach was similar to the classic anterior approach for the placement of anterior axis dens screws and the Minimal Exposure Tubular Retractor (METRx) was placed to maintain the surgical field. Although their proposed procedure had the advantage of avoiding traversing the oral, the limited, deep operative field, and inconvenient extra-long working distance made big challenge to surgeons.

Advance in endoscopic technology has allowed the appliance of endoscope in the odontoidectomy. Wolinsky et al. described a full endoscopic trans-cervical approach for odontoid resection (6). A modified METRx, the neural endoscope, and high-speed burr contributed to make a clear operative view and convenient procedure. But placing a retractor more than 2-cm diameter also accompanies the high risk of stretch injury of superior laryngeal nerve, hypoglossal nerve, and marginal branch of the mandibular nerve. Meanwhile, continuous bleeding of cancellous bone added much disruption in endoscopic procedures.

Recent progress in spinal endoscopy, especially the closed tubular sleeve that enables stop bleeding with water pressure and irrigation, integrated coaxial spinal endoscope system, and high-speed tip-changeable endoscopic burr, enables spine surgeon to perform bone resection and decompression of the occipitocervical region in a 6–7 mm tubular sleeve (7). Based on the accumulated experience in anterior cervical surgeries and percutaneous endoscopic lumbar discectomy (PELD), we developed a novel full-endoscopic trans-cervical odontoidectomy and medulla oblongata ventral decompression.

## Materials and methods

### Patient characteristics

This retrospective case series contains two patients undergoing the full-endoscopic trans-cervical odontoidectomy from 2018 to 2020 upon obtaining their signed and informed consent.

Case 1. A 62-year-old man with a history of severe neck pain for one month. Computed tomography (CT) and magnetic resonance (MR) scans of cervical spine demonstrated an osteolytic lesion of odontoid process combining pathological fracture. Positron emission tomography (PET) showed a single, 18 g-fdg high concentrated lesion in the odontoid process. Infection and septic markers were basically normal and no abnormality in tumor markers was noted. The Gram-stained smears from bone marrow aspiration suggested the plasmacytic myeloma. We considered that patient with odontoid process lesion and pathological fracture was candidate to perform odontoid process biopsy and resection, combining posterior C1–C3 instrumentations.

**Figure 1:**
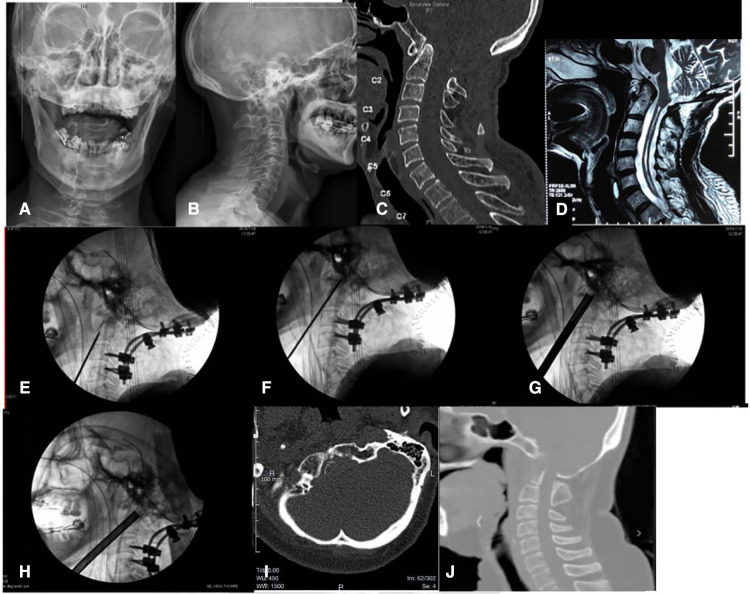
(A) AP x-ray of cervical spine of Case 2 before operation. (B) Lateral x-ray of cervical spine of Case 2 before operation. (C) Sagittal section of CT scan of Case 2 before operation. (D) Sagittal section of MR scan of Case 2 before operation. (E) The C-arm image when a Kirschner wire was drilled into the middle of the odontoid process of the axis in operation. (F) The C-arm image showed the depth of Kirschner wire reach the front of the odontoid process rear cortex and do not penetrate the rear cortex of the odontoid process. (G) The C-arm image showed a 7.2-mm hollow ring saw remove an annular bone block around the Kirschner wire. (H) The C-arm image showed lifting the end of the ring saw (the supine position) to make the angle between the ring saw and the axis of the spine larger, and removing more cancellous bone of the dentate bottom with the ring saw. (I) Cross section of CT scan after surgery. (J) Sagittal section of CT scan after surgery.

Case 2. A 70-years-old man was diagnosed as basilar invagination and had foramen magnum decompression surgery history 10 years ago. In the last 3 years, the weakness of his right limbs and the numbness of both lower limbs have gradually exacerbated. X-ray and CT scans showed that the odontoid process protruded into the foramen magnum (Figures 1A–C). MR showed medulla oblongata compression by odontoid process, combining with cervical syringomyelia (Figure 1D). Electromyography suggested that neurological impairments originated from upper spinal cord lesions, due to the obvious compression of medulla oblongata, which resulted in syringomyelia and neurological deficits. Resection of the odontoid process, the medulla oblongata decompression, and posterior occipitocervical fusion was required.

Case 3. A 54-year-old female with a history of numbness in her limbs for 3 years and inability to walk in her lower limbs for half a year. X-ray and CT scans showed that the odontoid process protruded into the foramen magnum. MR showed medulla oblongata compression by odontoid process, combining with cervical syringomyelia. Thus, resection of the odontoid process and medulla oblongata decompression was required.

### Surgical technique

After general anesthesia with tracheal intubation, the patient was positioned prone on the Jackson table, with somatosensory and motor evoked potential monitoring throughout the operation. Additional posterior instrumentations and fusion was performed to stabilize the spine before the odontoidectomy.

Subsequently the patient was placed in a recumbent position with a shoulder roll placed behind the neck to achieve gentle cervical extension. The head was fixed by a Mayfield head-holder. The operative area was prepped and draped in a standardized fashion for anterior cervical operations.

**Figure 2:**
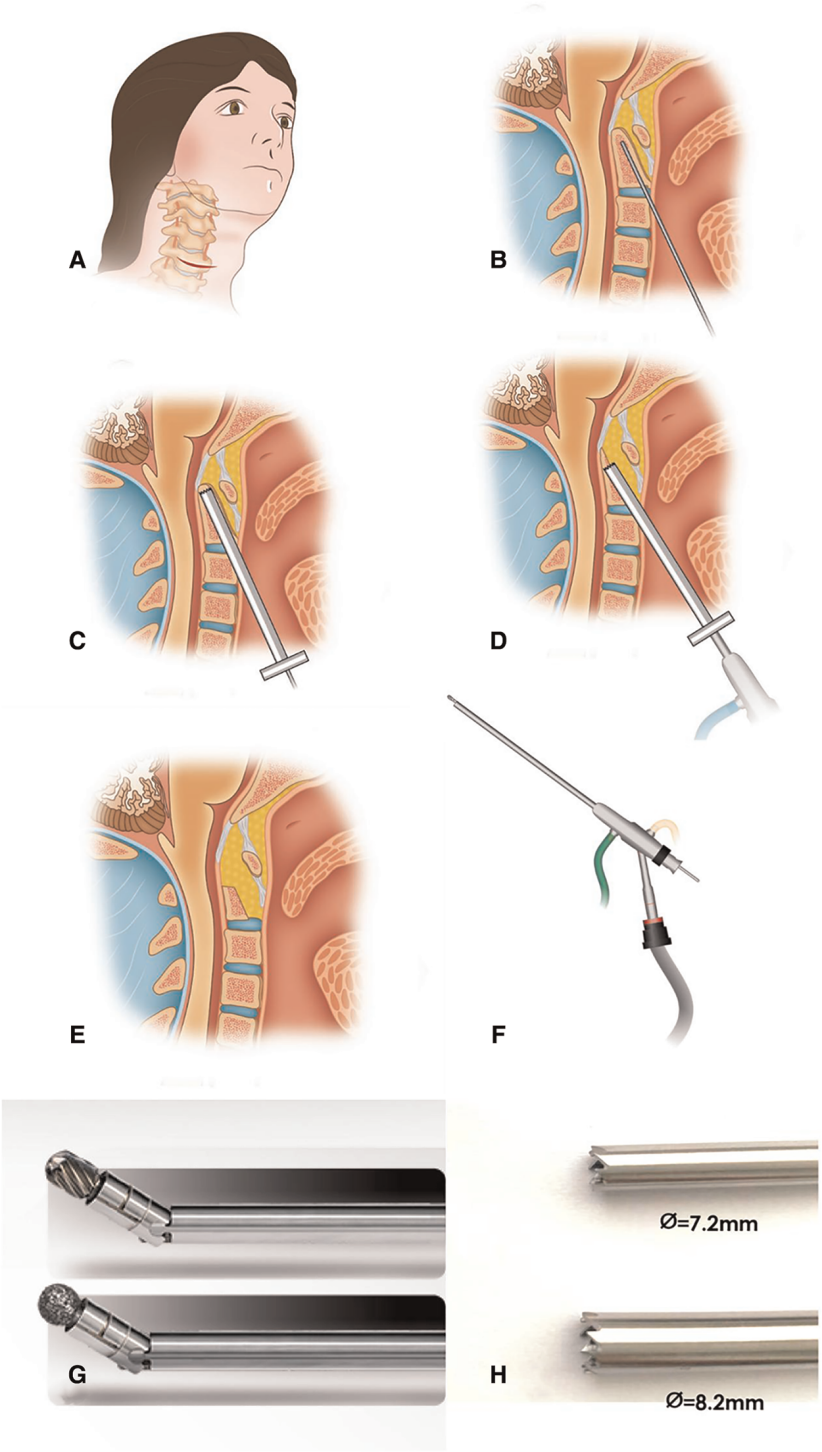
(A) A transverse incision was made at approximately the C-4 level on the right hand side of the patient, starting from the center line, extending to 3 cm in horizontal. (B) A Kirschner wire was drilled into the middle of the odontoid process of the axis. The position of the Kirschner wire was recommended to be located in center of the odontoid process in both AP and lateral x-ray. (C) A hollow ring saw was used to remove an annular bone block with the direction of the Kirschner wire. The hollow ring saw should also stop before odontoid process posterior cortex. (D) After removing the tip of the odontoid, simply lift the end of the ring saw (patient in the supine position) to make the angle between the ring saw and the axis of the spine larger and continue to remove more cancellous bone of the dentate bottom with the ring saw. (E) The odontoid process is removed and decompression of spinal cord is completed. (F) The 6.5-mm diameter coaxial spine endoscope. (G) The electric high-speed grinding drill, which is designed for endoscopy specifically and the direction of tip is changeable. (H) Feature of the tip of 7.2/8.2-mm hollow ring saw.

The standard Smith–Robinson approach was chosen for the access to cervical spine (8). A transverse incision was made at approximately C-4 level on the right side of the patient, starting from the central line, horizontally extending to right side about 3-cm length (Figure 2A). Dissect the subcutaneous tissue and platysma muscle. The esophagus and trachea were swept medially; the sternocleidomastoid muscle, carotid sheath, and the areolar tissue were swept laterally by blunt dissection. The spine was exposed rostrally to the anterior tubercle of atlas. Under fluoroscopy guidance, a Kirschner wire was drilled from the base of the dens, then cranially track to the tip of odontoid process. The position of the Kirschner wire was recommended to be located in the center of the dens, verified by fluoroscopic images on two orthogonal planes (Figures 1E, 2B). The rostral tip of Kirschner wire should keep close but in front of dorsal cortex of odontoid process. Penetration of the dorsal cortex must to be avoided (Figure 1F). Then a 7.2-mm hollow ring saw (Figures 1G, 2C) was applied to remove an annular bone block around the Kirschner wire and the resection of bone block should progress carefully without breaching to the dorsal cortex. Slightly rotation of the hollow ring saw in situ can separate the front part of the annular bone block, which will be taken out as the ring saw pulled out. A 7.2-mm, circular osseous space was built in the central part of odontoid process, then a 7.2-mm endoscopic working sleeve was inserted into the circular osseous space. A 6.5-mm coaxial spine endoscope was placed in the working sleeve. Continuous water irrigation was needed to stop bleeding of cancellous bone and maintain the clarity of the endoscopic vision during the procedure. The tip of the odontoid process was gradually resected from the inside to outside by an electric high-speed burr, which was especially designed for endoscopy, and the angle of the burr's tip was adjustable from 0° to 45° (Figure 2G). After removing the cranial part of the dens, backed off the endoscopic sleeve but no exit completely from the bone tunnel. Then re-inserted the ring saw and tilted the end of the ring saw (the supine position) to increase the angle between the ring saw and the odontoid process, and removed more cancellous bone of the odontoid process's bottom (Figures 1H, 2D). Because the bottom portion of the odontoid process was little wider, a larger diameter sleeve and ring saw can be chosen in this procedure (Figure 2H), which can improve the efficiency of bone resection and decompression. The decompression of the medulla oblongata was completed until all the ventral bone was removed and the alar ligaments was exposed; however, the dura need not to be exposed (Figure 2E).

Because it is difficult to determine the range of the odontoid resection and medulla oblongata decompression under endoscope, the O-arm scan was necessary to judge whether the range of odontoid resection and decompression was enough.

## Results

Patients were extubated soon after surgery. Continuous monitoring of pulse oxygen saturation at fingertip showed 100%. They were able to normally vocalize and communicate without complaining of discomfort such as dyspnea and dysphagia. Without the need for nasogastric tube placement, oral feeding can be resumed at 6 h postoperatively, no coughing caused by feeding or dysphagia reported.

In Case 1, the VAS score was two points at third days postoperative and improved significantly compared with nine points preoperative. Immunohistochemical staining of the extracted sample confirmed the diagnosis of plasmacytoma. The patient received local radiotherapy 3 weeks after operation and still under follow-up.

In Case 2, the patient claimed that the numbness of lower limbs and the weakness of right limbs significantly release at a week postoperatively. At 6-month's follow-up, Japanese Orthopedic Association (JOA) score increased from 7 preoperatively to 18 postoperatively, with no progression of myelopathy symptoms.

In Case 3, the patient claimed that the numbness of her limbs and the weakness of lower limbs significantly release at two week postoperatively. At 6-month's follow-up, Japanese Orthopedic Association (JOA) score increased from 6 preoperatively to 15 postoperatively, with no progression of myelopathy symptoms.

The postoperative CT scans displayed the extent of odontoid process resection consistent with the pre-operative expectation (Figures 1I,J).

## Discussion

Nowadays, the trans-oral approach has been the benchmark for odontoidectomy (1, 2). With the advent of its various surgical modifications (e.g., an extended trans-maxillary, maxillary split, trans-palatal, or trans-mandibular approach), these practices are noted to be complicated by phonation dysfunction and velopharyngeal insufficiency as well as cosmetic deformity (9). In addition to the limited operative view, the deep location and complex surrounding structures that hinder the operated structures, the trans-oral approach is highly complicated by bacterial contamination from flora in the oropharynx, prolonged intubation or tracheostomy due to swelling of soft tissue, and pharyngeal wound dehiscence that requires nasogastric tube feeding (10). Patients with severe oral cavity deformities like micrognathia are not suitable candidates for this procedure.

Alternative approaches were implemented to decrease the complications related to approach in conventional transoral procedure. Among alternative approaches, studies on retropharyngeal technique were sporadically reported. Fong and DuPlessis have developed a retropharyngeal exposure to the odontoid process in the cadaver (5). They used the METRx as retractor and working channel. Wolinsky et al. reported a similar surgical approach with endoscopic assisted to perform the retropharyngeal odontoidectomy and brainstem decompression without traversing the oral cavity (6). In their practice, endoscopic odontoidectomy was mainly performed through modified METRx, which was more than 2 cm in diameter. Compared with Wolinsky's method, our endoscopic trans-cervical odontoidectomy has some advantages. First, the diameter of hollow ring saw and endoscopic retractor was no more than 8.2 mm and the exposure was similar to placing the axial odontoid screw, which has been proved to be safe and feasible. The choice of 7.2-mm or 8.2-mm ring saw was made upon personalized size of the odontoid process. In Wolinsky's method, the operation was performed through a retractor which diameter is more than 2 cm. It needs a much larger exposure than us, and the superior laryngeal nerve, hypoglossal nerve along with marginal branch of the mandibular nerve were at higher risk of a stretch injury. Second, the technique reported by Wolinsky was susceptible to possible tubular retractor displacement resulting in range deviation of the surgical resection and accidentally injuring the surrounding structure. In our method, the 7.2-mm endoscopic sleeve was embedded into axial vertebrae and fixed by the circular bone. It has very few opportunity of sleeve displacement. Therefore, there is no need for external fixtures to restrain the displacement of the retractor. Third, Wolinsky's endoscope is more similar to MED (micro endoscopic discectomy), occasionally bleeding due to the paravertebral veins and cancellous bone marrow would blur the surgical vision. We performed this operation with a 6.5-mm coaxial spine endoscope, which is frequently applied in percutaneous lumbar transforaminal endoscopic discectomy. Continuous water irrigation and water pressure guaranteed a clear vision during endoscopic operation.

Ruetten et al. performed the similar procedure as us to treat infections of the anterior craniocervical junction (11–13). In their report, sufficient decompression and debridement resulted rapid regression of the clinical and neurological symptoms, as well as healing of the infection. In the report, they claimed that the sign of free floating of dura mater in the irrigation fluid indicated effective decompression. Our method had some similarities with Ruetten's method. The two methods both had the trans-cervical approach, both performed the operation with coaxial spine endoscope and have continuous water irrigation guarantees to stop bleeding. Additionally, because this is a typical full-endoscopic uniportal approach, other common technology for bleeding control also be used in our approach including controlled hypotension and radiofrequency ablation. But we also had some improvement compared with Ruetten's. First, in Ruetten's method, the working sleeve is placed in front of the odontoid process and outside of the bone, and the odontoid process was totally resected from the ventral to dorsal. In our method, a uniportal working sleeve was placed inside of the odontoid process with fluoroscopic guidance, then odontoid process was gradually resected from the inside to outside with the help of electric high-speed burr, which was newest designed for endoscopy and the tip is adjustable from 0° to 45° (Figure 2G). It should be safer that the resection of odontoid process from the inside to outside. Second, in Ruetten's method, the endoscopic sleeve was removable to achieve extent the range of resection and decompression. In our method, endoscopic working sleeve was embedded into axial vertebrae and fixed by the circular osseous space. With the help of special burr to extend the range of resection and decompression, the uniportal spinal endoscope has a very high magnification and accompanies a very limited version, so it is difficult to identify anatomical structure and locate the operation site *via* the view gotten from the monitor. If the sleeve was lack of effective fixation, deviation of the operative site from the target area may not to be realized in time. With fluoroscopic guidance, endoscopic sleeve is embedded into axial vertebrae and fixed by the circular osseous space could avoid deviation from the target area and repeatedly fluoroscopic position. Third, we had the hollow ring saw to remove the majority of the odontoid process, and it is more efficient than burr. The tip changeable burr enlarges the range of osteotomy and decompression on the basis of circular saw.

Additionally, Yukoh Ohara et al. also performed the similar procedure as us for odontoidectomy (14). Both of us used an “inside to outside” approach for odontoidectomy. However, there are still some differences between our approaches. In our approach, we first used a 7.2-mm hollow ring saw to remove most of the odontoid process whereas Yukoh Ohara et al. conducted the whole odontoidectomy throughout the drill. Thus, we believe our approach should be more efficient. Moreover, similar to Ruetten's approach, they need constant fluoroscopy to identify anatomical structures and avoid displacement of the working sleeve, while in our approach, the endoscopic sleeve is embedded into axial vertebrae and fixed by the circular osseous space which can avoid deviation from the target area and repeatedly fluoroscopic position. In summary, we believe Yukoh Ohara's approach is more like Ruetten's than ours.

However, there are some notable disadvantages associated with the full-endoscopic trans-cervical odontoidectomy we proposed. First, the bone resection and decompression was mainly limited to odontoid process, so only odontoid process biopsy and resection could be performed in case. Other procedures such as loosening and integration the lateral joint of axis and atlas seem to be difficult to perform with our method. Second, due to current equipment limitations, it is impossible to implement ventral supportive bone grafting and anterior instrumentation for axis and atlas. In addition, it is difficult to judge the actual extent of resection visually in the view of the endoscope, which in turn necessitates the use of the O-arm or C-arm for confirmation. The obese and the patients with severe cervical spine deformity also need to be excluded. Additionally, more patients treated with this technique with longer follow-up is warranted to confirm the feasibility and validity.

## Conclusion

When performing simple odontoid resection, the proposed endoscopic trans-cervical odontoidectomy provides a valid choice for non-oral approach, which would reduce postoperative approach-related complications and accelerate postoperative recovery. But it needs a surgeon to be highly experienced in endoscopic procedures, and the range of surgery is mainly restricted to odontoid processes.

## Data Availability

The raw data supporting the conclusions of this article will be made available by the authors, without undue reservation.
